# Turner syndrome presenting with idiopathic regression: A case report

**DOI:** 10.1111/pcn.13483

**Published:** 2022-10-20

**Authors:** Kosuke Mizobuchi, Itaru Kushima, Hidekazu Kato, Mari Miyajima, Hiroyuki Kimura, Norio Ozaki

**Affiliations:** ^1^ Department of Psychiatry Nagoya University Hospital Nagoya Japan; ^2^ Department of Psychiatry Nagoya University Graduate School of Medicine Nagoya Japan; ^3^ Medical Genomics Center Nagoya University Hospital Nagoya Japan; ^4^ Institute for Glyco‐core Research (iGCORE) Nagoya University Nagoya Japan

Turner syndrome (TS) is caused by either the complete or partial loss of one X chromosome, often in mosaic karyotypes. Patients with TS have a higher incidence of intellectual disability, autism spectrum disorder (ASD), attention‐deficit/hyperactivity disorder, schizophrenia, and eating disorders.^1^, ^2^ Here, we report the first case of a patient with TS who showed idiopathic regression with loss of previously acquired adaptive, cognitive, and social functioning.

The patient, a 25‐year‐old Japanese woman, was born at 40 weeks with a birth weight of 3590 g. Her early developmental history was unremarkable except for short stature. In elementary school, she attended regular classes, participated in club activities, and socialized with her friends in a normal way. Her academic performance was average except for arithmetic. She had difficulties mastering number sense and calculation and used fingers to add single‐digit numbers (selective learning disability). At the age of 9 years, TS was suspected based on her short stature (113.6 cm, −2.3 SD). Karyotype analysis confirmed the diagnosis of TS. She received growth hormone therapy. After graduating from high school, she started working. When she was in her early 20s, her grandfather passed away. She then began to speak and make eye contact less frequently and was no longer able to take care of herself. Her behavior became disorganized and disinhibited. She was suspected of having schizophrenia and was started on aripiprazole (3 mg/d), but her condition did not improve and her deviant behaviors (e.g. leaving a restaurant without paying) continued. She also had episodes of overeating that were troubling to her family (unspecified eating disorder) and became obese (body mass index, 36.5 kg/m^2^), eventually developing diabetes. At the age of 24 years, she was admitted to a psychiatric hospital. While in the hospital, she rarely spoke, had scant facial expressions, and required careful observation because of her disorganized behavior. Delusions, hallucinations, and disorganized speech were not present throughout her clinical course. Her IQ was 27 on the Tanaka‐Binet Intelligence Scale‐Fifth Edition. The characteristic symptoms of TS, including amenorrhea, cubitus valgus, excessive pigmented nevi, short fourth finger and toe, and spoon‐shaped nails, were noted. Blood tests, brain magnetic resonance imaging (MRI), and electroencephalogram did not reveal the cause of the regression. Brain MRI showed hypoplasia of the corpus callosum and an arachnoid cyst (Fig. S1), which have been reported in TS.^3^ Array comparative genomic hybridization indicated mosaic X chromosome monosomy (Fig. 1a). The result of a G‐banding test was 45,X[18]/46,X,i(X)(q10)[12], indicating mosaicism of 45,X and a structurally abnormal X chromosome consisting of two long arms (46,X,i(X)(q10)) (Fig. 1b). She did not meet the diagnostic criteria for ASD, schizophrenia, or mood disorder. Her condition was considered idiopathic regression, as seen in Down syndrome (DS).^4^, ^5^

**Fig. 1:**
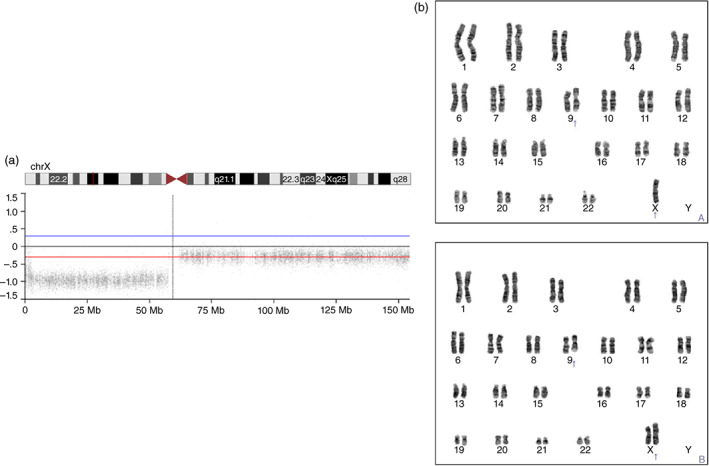
Results of genetic testing. (a) Array comparative genomic hybridization shows that the signal values (log2Ratio) were −1 for the short arm of the X chromosome and −0.3 for the long arm, suggesting the mosaic karyotype of Turner syndrome. (b) Results of a G‐banding test. The upper and lower panels show 45,X and 46,X,i(X)(q10) karyotypes, respectively.

After discharge, she was followed up without any medication. Her disorganized behavior became increasingly worse and her family could no longer cope. She was treated with low doses of antipsychotics, but they were not effective. Therefore, she was readmitted to a psychiatric hospital. She demonstrated problematic behavior such as going into other patients' rooms and not following the nurses' instructions. Administration of blonanserin (24 mg/d) reduced some of these behaviors.

Soon after discharge, she developed a fever and gradually became immobile and unresponsive. A polymerase chain reaction test confirmed the diagnosis of coronavirus disease 2019 (COVID‐19). She was in a state of substupor and exhibited catalepsy, maintaining her upper extremities in a bizarre position. She was diagnosed with catatonia and hospitalized. Lorazepam (1.5 mg/d) improved her catatonia and regression in 2 weeks. After discharge, she remained stable while taking lorazepam and blonanserin and was able to have conversations with others, help with household chores, and cook for her family.

Idiopathic regression is relatively frequent in DS (trisomy 21) and is referred to as Down syndrome disintegrative disorder (DSDD).^4^, ^5^ Common features of regression shared by the present case and patients with DSDD are onset in young adulthood, the presence of potential triggering life events or environmental changes, catatonia, and effectiveness of benzodiazepines. The present patient became catatonic after being infected with severe acute respiratory syndrome coronavirus 2. Infectious diseases including COVID‐19 are reported to cause catatonia.^6^, ^7^ There are potential mechanisms for this: viral infiltration into the central nervous system, cytokine network dysregulation, peripheral immune cell transmigration, and postinfectious autoimmunity.^8^ The present case is valuable because it shows that idiopathic regression as seen in DS can also occur in TS. However, more cases should be accumulated to clarify this association.

## Disclosure statement

K.M., I.K., H.Ka., M.M., and H.Ki. declare no conflicts of interest. N.O. has received research support or speakers' honoraria from, or has served as a consultant to, Sumitomo Dainippon, Eisai, Otsuka, KAITEKI, Mitsubishi Tanabe, Shionogi, Eli Lilly, Mochida, DAIICHI SANKYO, Nihon Medi‐Physics, Takeda, Meiji Seika Pharma, EA Pharma, Pfizer, MSD, Lundbeck Japan, Taisho Pharma, Sumitomo Pharma, TSUMURA, Kyowa Kirin, and Viatris, outside the submitted work.

## Ethical approval

This study was approved by the ethics committee of the Nagoya University Graduate School of Medicine and complied with all the provisions of the Declaration of Helsinki.

## Consent to participate

The patient and her family gave written informed consent for the genetic analysis and publication of this report.

## Supporting information


**Figure S1**: Brain MRI of the patient.
