# Supplementary figures and images for: Turner syndrome presenting with idiopathic regression: A case report
Source: Psychiatry Clin Neurosci. 2022 Oct 20;76(12):680–2. doi: 10.1111/pcn.13483 (PMC10092704; doi:10.1111/pcn.13483)

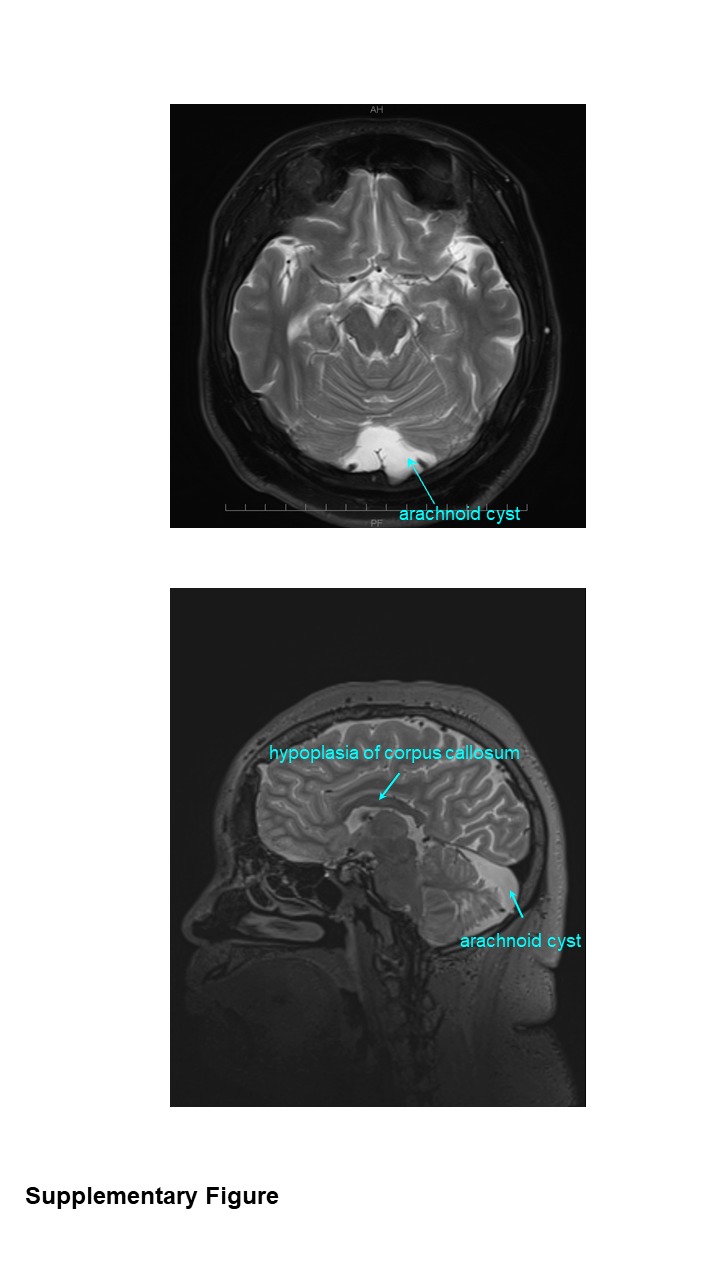

Supplement: Supplementary file 1 — Figure S1: Brain MRI of the patient. [file PCN-76-680-s001.png]
